# The Effect of Ethanol on the Release of Opioids from Oral Prolonged-Release Preparations

**DOI:** 10.1080/03639040701377292

**Published:** 2007-10-25

**Authors:** Malcolm Walden, Fiona A. Nicholls, Kevin J. Smith, Geoffrey T. Tucker

**Affiliations:** Mundipharma Research Limited, Cambridge Science Park, Milton Road, Cambridge, UK; Academic Unit of Clinical Pharmacology, University of Sheffield, The Royal Hallamshire Hospital, Sheffield, UK

**Keywords:** prolonged-release, opioid, hydromorphone, in vitro dissolution rate, ethanol (or alcohol) testing, pharmacokinetics

## Abstract

Recent experience has prompted the US FDA to consider whether ethanol ingestion may modify the release characteristics of prolonged-release formulations, where dose dumping may be an issue for patient safety.

The influence of ethanol on the in vitro release of opioid drugs from some prolonged-release formulations utilizing different release technologies was examined. Results indicated that the prolonged-release mechanisms remained intact under the testing conditions, although one product showed initial sensitivity to ethanol in its release characteristics. Nevertheless, in this case, extrapolation of the findings to likely outcome in vivo indicated no risk of dose-dumping.

It is proposed that prolonged-release medicinal products should be tested during development to ensure robustness to the effects of ethanol on drug release.

## INTRODUCTION

Since their introduction in the 1980's, oral prolonged-release opioid formulations have been a mainstay in the management of moderate to severe pain, particularly that due to cancer. *MST*® *Continus*® tablets were introduced to provide a controlled delivery of morphine over 12 h. This was followed by 12 h release formulations of hydromorphone (*Palladone*® SR capsules), oxycodone (*OxyContin*® tablets), dihydrocodeine (*DHC*® *Continus* tablets), and tramadol (*Zydol*® SR tablets, *Dromadol*® SR tablets, and *Zamadol* SR capsules). Subsequently, preparations providing the delivery of morphine (*MXL*® capsules, *Morcap* SR capsules) and tramadol (*Zydol* XL tablets) over a 24 h period were developed, enabling the management of pain with a convenient once-daily dosing regimen.

As these formulations contain a larger unit dose than conventional, immediate release tablets, which are intended for 4–6 hourly administration, it is imperative that their retardation properties are tightly controlled. This is to ensure that a rapid release of the drug (“dose-dumping”) cannot occur. Prolonged-release formulations are subject to stringent limits on in vitro dissolution to ensure a consistent batch-to-batch uniformity of the finished product. In addition, studies are conducted in healthy volunteers to investigate the influence of a high-fat meal, and the associated delayed gastric emptying, on the in vivo release characteristics of each formulation, to ensure that the prolonged delivery of the active moiety is maintained, regardless of dietary status.

**FIGURE 1:**
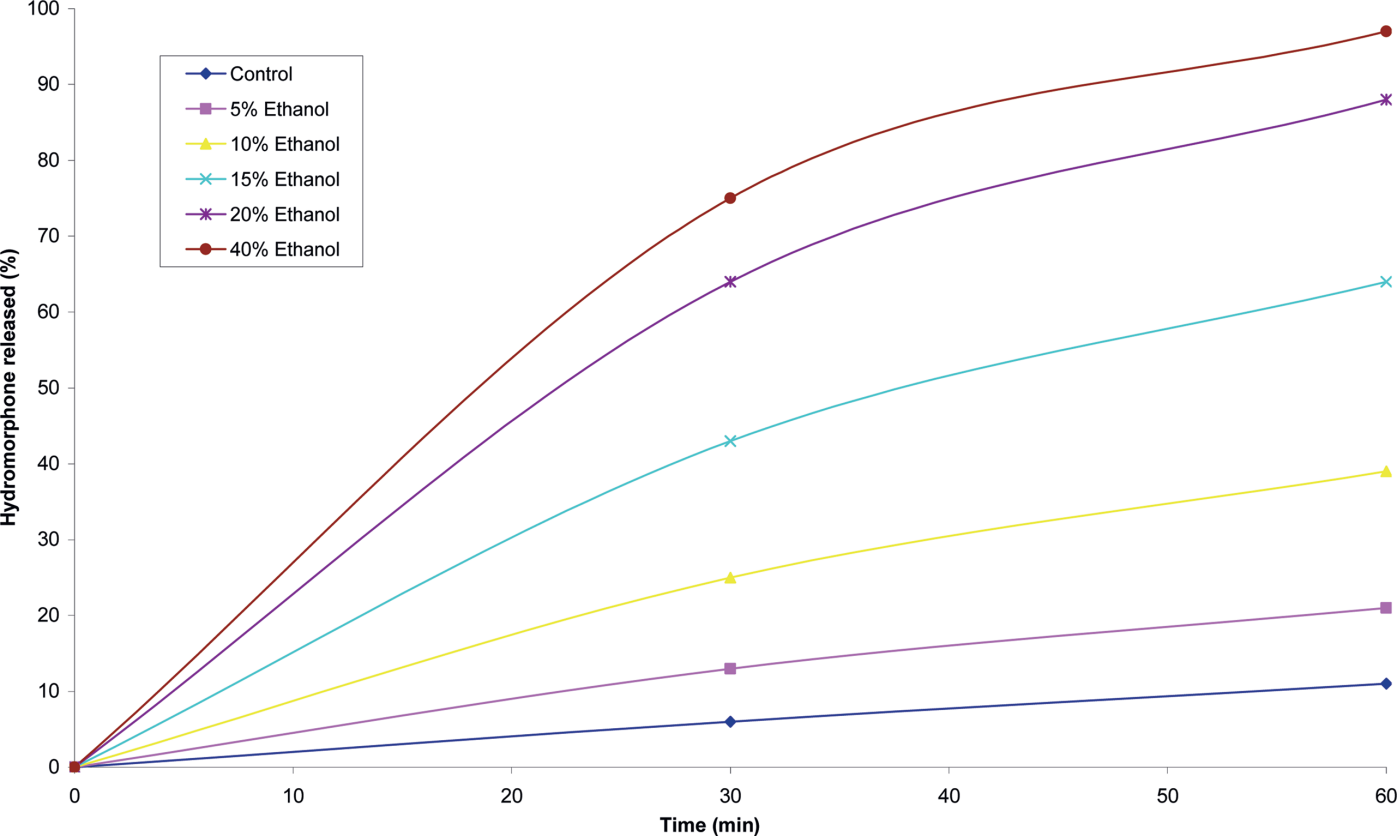
Cumulative release of hydromorphone from hydromorphone once daily capsules in simulated gastric fluid/ethanol.

Some oral prolonged-release dosage forms contain drugs and excipients that exhibit a higher solubility in aqueous solutions containing ethanol. Accordingly, such products may be expected to exhibit more rapid drug dissolution and release in the presence of ingested ethanol. Recently, a once-daily formulation of hydromorphone was withdrawn from the US market following concern over the potential for ethanol-induced dose-dumping. The manufacturers had been prompted to conduct a pharmacokinetic study by the results of an in vitro evaluation of the dissolution of the preparation in different concentrations of ethanol. The results of the latter evaluation are presented in Figure 1, showing a more rapid release of hydromorphone in increasing concentrations of ethanol in aqueous solution.

In a subsequent pharmacokinetic study, healthy subjects were protected from the effects of the opioid agonist by naltrexone blockade (HMP1013). Each subject received a single prolonged-release capsule, containing 12 mg of hydromorphone hydrochloride, according to a randomised, crossover design, including a control arm (no ethanol) and 3 arms involving the ingestion of 240 mL of varying concentrations of ethanol immediately before the once-daily hydromorphone prolonged-release capsule. The two higher concentrations of ethanol represented the equivalent of drinking one-third of a bottle of spirit or one-third of a bottle of fortified wine, respectively, over an approximately 5 min period. The results are summarised in Table 1.

**Table 1:** The Effect of Different Concentrations of Ethanol on Systemic Exposure to Hydromorphone After Administration of 12 mg in Hydromorphone Once-Daily Capsules

Ethanol Concentration (% v/v)	Mean *C*_max_ Ratio (and range) (Relative to Control No Ethanol)	Mean AUC Ratio (and Range) (Relative to Control No ethanol)
40	5.53 (0.77–15.8)	1.26 (0.61–3.35)
20	1.89 (0.76–5.72)	0.96 (0.41–1.46)
4	1.06 (0.73–1.96)	1.00 (0.48–1.85)

*C*_max_=maximum plasma drug concentration; AUC=total area under the plasma drug concentration—time curve.

On average, there was a 26% increase in the AUC of hydromorphone when administered following the ingestion of 40% ethanol. The maximum systemic exposure, as indicated by C_max_ values, and, therefore, the rate of hydromorphone absorption, was increased markedly in some individuals, particularly at the higher concentration of ethanol. After the ingestion of 40% ethanol, the mean increase in C_max_ was 5.5-fold, with one individual showing a 16-fold increase.

These findings were confirmed in a subsequent study (HMP1014), which also showed that the ingestion of ethanol either 3 h before or 4 h after the administration of the once-daily prolonged-release hydromorphone capsule had no effect on the kinetics of the opioid, thereby providing guidance on the time period within which the drug preparation and ethanol should not be taken together.

It was recognised that the most extreme test treatment, equating to ingestion of up to the equivalent of one-third of a bottle of spirit taken over only 5 min, represented an extreme challenge to the formulation. Nevertheless, the once-daily prolonged-release hydromorphone capsule was withdrawn voluntarily by the manufacturer, removing any potential risk to patients of inadvertent dose-dumping.

This recent experience has prompted the US FDA to consider further the issue of whether ethanol ingestion may modify the release characteristics of new and currently marketed prolonged-release formulations. The main objective of such an approach will be to minimize the risk of ethanol-induced dose dumping, irrespective of any warnings on product labelling and instructions by health care providers (Meyer & Hussain, 2005). Clearly, the risk is accentuated for products containing drugs that have a narrow therapeutic margin, or those containing drugs such as potent opioids where careful titration of dose to effect is necessary.

However, evaluating the consequences of co-ingestion of high doses of ethanol together with prolonged-release formulations in healthy subjects is also not without risk, even in the case of opioids where the co-administration of an opioid antagonist is also feasible. A potential regulatory approach could be to require a standard series of in vitro dissolution tests to establish that the drug-release characteristics of the originator product or generic product are robust in the presence of ethanol.

Against this background, the present investigation examined the influence of high concentrations of ethanol on the in vitro release properties of a range of prolonged-release opioid formulations which utilise a variety of different release technologies (matrix control (monolithic and multiparticulate), ion-exchange, coated beads).

## METHODS

The effect of ethanol on drug release from the products listed in Table 2 was evaluated by the corresponding dissolution tests used routinely for quality control. Some of these tests simulate stomach pH while others simulate the average pH in the small intestine. Standard dissolution testing equipment defined in pharmacopoeial monographs was used. The dissolution media were modified by replacing appropriate volumes of the standard aqueous media with volumes of ethanol to produce solutions with ethanol concentrations up to 40% v/v. This upper limit was chosen as it represents a standard strength of spirits. It is recognized that, in vivo, the ingestion of spirits in such concentration will be subject to immediate dilution by the residual volume of the stomach (typically 50 mL in the fasting state) with further dilution over time due to gastric secretion, the intake of food and fluid, and the absorption and metabolism of ethanol in the stomach. Thus, in vitro experiments with concentrations of 40% ethanol over a 2 h period are expected to be representative of the most extreme conditions likely to be encountered in vivo.

**Table 2:** Products Details

	Active Ingredient	Formulation Technology/Excipients	Dissolution Medium	Dissolution Method
*DHC*® *Continus*® 120 mg tablets*	Dihydrocodeine tartrate	CONTINUS® matrix control	Phosphate buffer pH 6.5 Ph. Eur. For each litre dissolve in deionised water: 6.805 g potassium dihydrogen orthophosphate (AR), 0.56 g sodium hydroxide (AR). Purge with helium; pH to 6.5 ± 0.05.	Paddles 100 ± 4 rpm 900 mL 37°C
		Lactose, cetostearyl alcohol, talc, hydroxyethylcellulose, magnesium stearate		
*MST*® *Continus*® 200 mg suspension*	Morphine sulphate	Controlled (Ion exchange) release granules in sachets	Modified USP simulated gastric fluid (no pepsin). For each litre dissolve 2.0 G of sodium chloride (AR) in 500 mL of deionised water. Add 7.0 mL of concentrated hydrochloric acid, dissolve and make to 1 litre with deionised water. Purge with helium; pH to 1.1 ± 0.05.	Paddles 100 ± 4 rpm 900 mL 37°C
		Cationic exchange resin, xanthan gum, Ponceau 4R (E124), xylitol, raspberry flavour		
*MST*® *Continus*® 100 mg tablets*	Morphine sulphate	CONTINUS® matrix control	Phosphate buffer pH 6.5 Ph. Eur. For each litre dissolve in deionised water: 6.805 g potassium dihydrogen orthophosphate (AR), 0.56 g sodium hydroxide (AR). Purge with Helium; pH to 6.5 ± 0.05.	Paddles 100 ± 4 rpm 900 mL 37°C
		Lactose, cetostearyl alcohol, talc, hydroxyethylcellulose, magnesium stearate		
*MXL*® 200 mg capsules*	Morphine sulphate	Matrix prolonged-release multiparticulates	Phosphate buffer pH 6.5 Ph. Eur. For each litre dissolve in deionised water: 6.805g potassium dihydrogen orthophosphate (AR), 0.56g sodium hydroxide (AR). Purge with Helium; pH to 6.5 ± 0.05	Paddles 100 ± 4 rpm 900 mL 37°C
		Hydrogenated vegetable oil, polyethylene glycol, magnesium stearate, talc		
*OxyContin*® 80 mg tablets*	Oxycodone hydrochloride	ACROCONTIN® matrix control	Modified USP simulated gastric fluid (no pepsin). For each litre dissolve 2.0 g of sodium chloride (AR) in 500 mL of deionised water. Add 7.0 mL of concentrated hydrochloric acid; dissolve and make to 1 litre with deionised water. Purge with Helium; pH to 1.2 ± 0.1.	USP Apparatus 1 (Basket) 100 ± 4 rpm 900 mL 37°C
		Lactose monohydrate, glyceryl triacetate, talc, ammoniomethacrylate polymer, povidone, stearyl alcohol, magnesium stearate		
*Palladone*® SR 24 mg capsules*	Hydromorphone hydrochloride	Coated bead technology	0.1% w/v sodium lauryl sulphate. For each litre dissolve 1 g of sodium lauryl sulphate (99% Sigma). Purge with Helium	Ph Eur Basket 150 ± 6 rpm 900 mL 37°C
		Microcrystalline cellulose, hydroxypropylmethyl cellulose, water, ethylcellulose, dibutyl sebacate		
Codeine *Contin*® 100 mg tablets**	Codeine base, Codeine sulphate	CONTINUS® matrix control	Purified, deionised water	USP Apparatus 1 (Basket) 100 ± 4 rpm 900 mL 37°C
		Lactose, stearyl alcohol, talc, hydroxyethylcellulose, magnesium stearate		
Zamadol® 24 h 400 mg tablets (once daily)***	Tramadol Hydrochloride	Matrix prolonged-release tablet	Phosphate buffer pH 6.5 Ph. Eur. For each litre dissolve in deionised water: 6.805g potassium dihydrogen orthophosphate (AR), 0.56g sodium hydroxide (AR). Purge with Helium; pH to 6.5 ± 0.05	Paddles 100 ± 4 rpm 900 mL 37°C
		Hydrogenated vegetable oil, talc, magnesium stearate		
Dromadol® SR 200 mg tablets (twice daily)****	Tramadol Hydrochloride	Matrix prolonged-release tablet	Phosphate buffer pH 6.5 Ph. Eur. For each litre dissolve in deionised water: 6.805g potassium dihydrogen orthophosphate (AR), 0.56g sodium hydroxide (AR). Purge with Helium; pH to 6.5 ± 0.05	Paddles 100 ± 4 rpm 900 mL 37°C
		Hydrogenated vegetable oil, talc, magnesium stearate		

* Marketed in the UK by Napp Pharmaceuticals Limited.

** Marketed in Canada by Purdue Pharma Inc.

*** Marketed in the UK by Meda Pharmaceuticals.

**** Marketed in the UK by Teva Pharma.

To offset the effects of chromatographic differences and different rates of evaporation of the modified media, standards used to assay the drugs were maintained at 37°C and prepared with the same media as used in each dissolution test. At least two representative unit doses of each formulation were tested in each case and the mean values of cumulative release were calculated. Drug concentrations were measured by reversed phase HPLC with UV detection at an appropriate wavelength. All of the drug assays were validated to ICH guidelines for specificity, linearity, accuracy and precision.

## RESULTS

**FIGURE 2:**
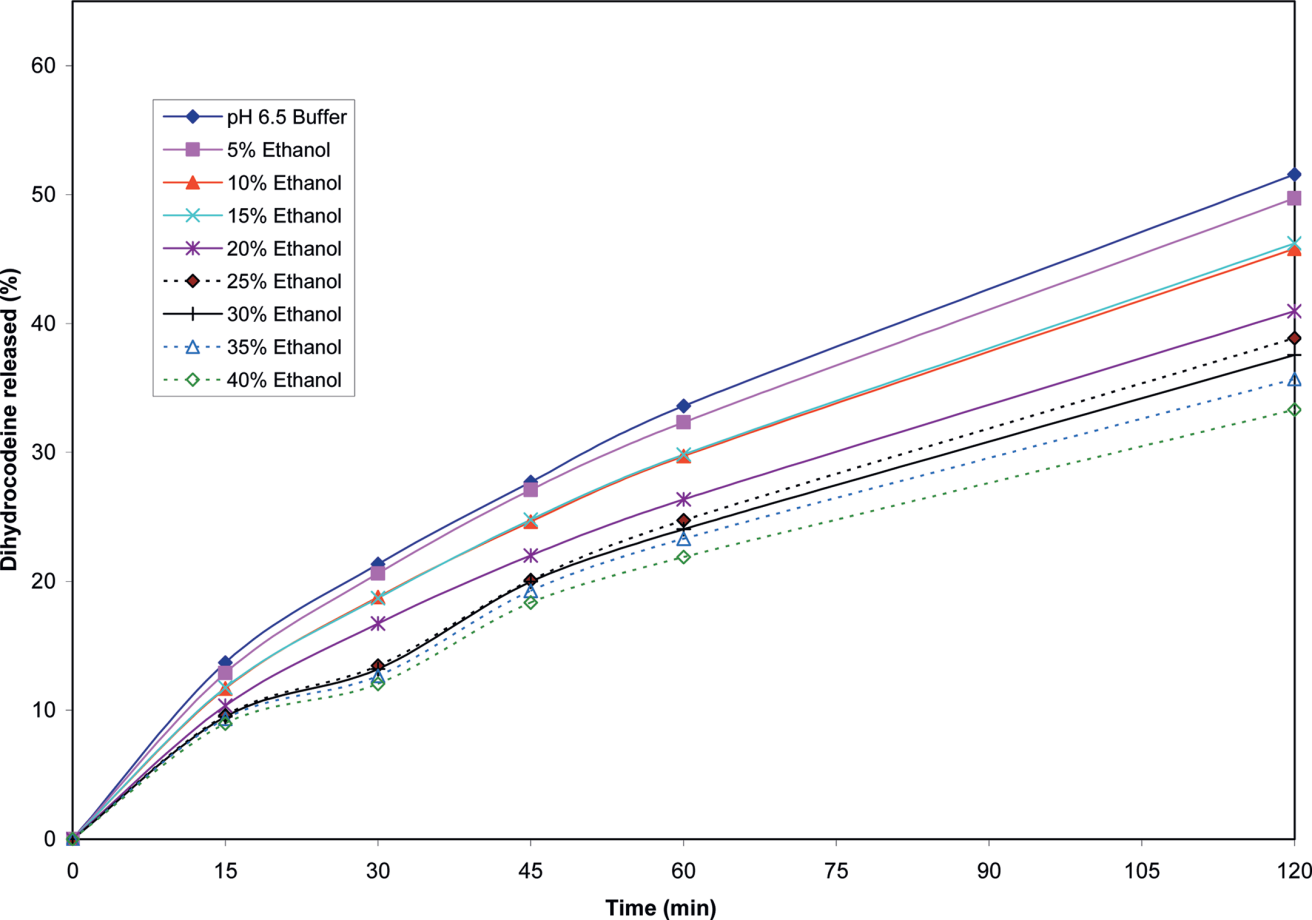
Cumulative release of dihydrocodeine from 120 mg DHC Continus tablets in pH 6.5 phosphate buffer/ethanol.

**FIGURE 3:**
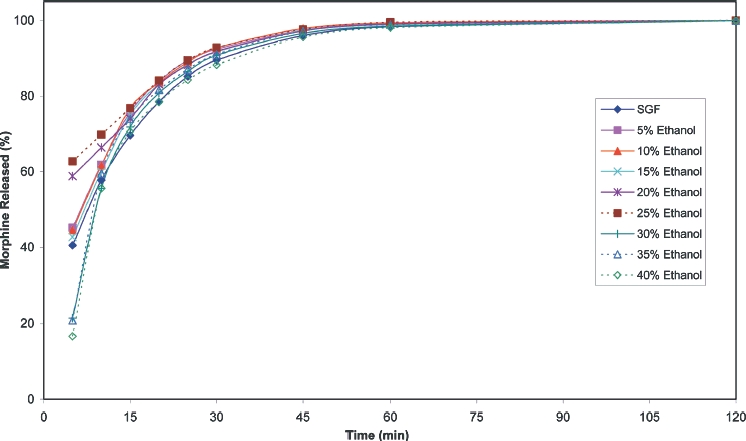
Cumulative release of morphine from 200 mg *MST Continus* suspension in simulated gastric fluid/ethanol.

**FIGURE 4:**
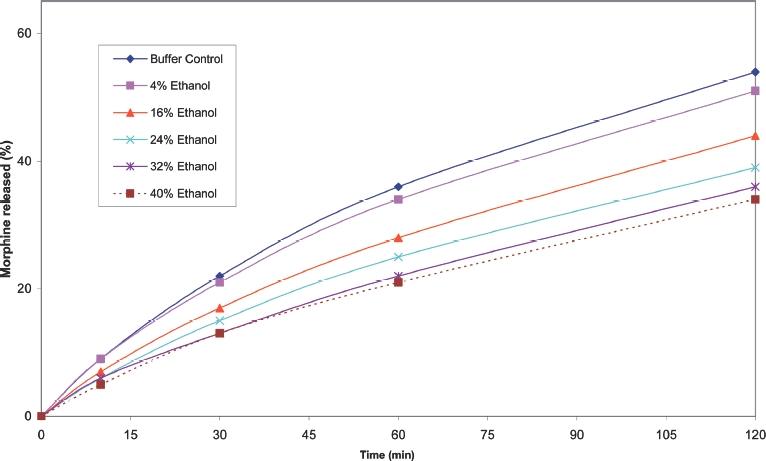
Cumulative release of morphine from 100 mg *MST Continus* tablets in simulated gastric fluid/ethanol.

**FIGURE 5:**
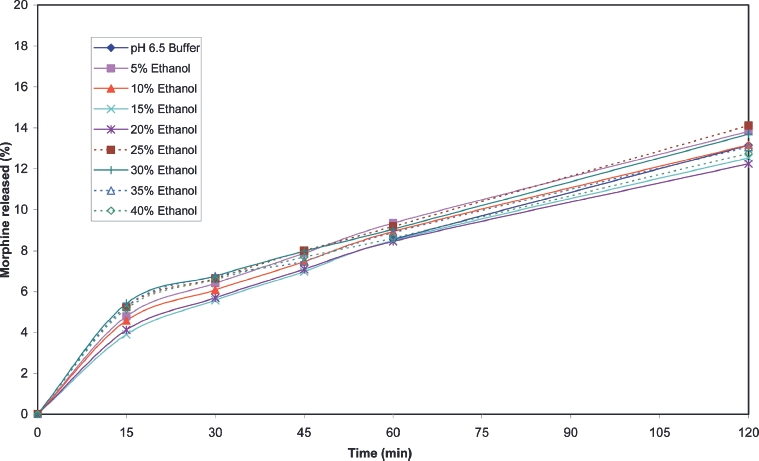
Cumulative release of morphine from 200 mg *MXL* CR capsules in phosphate buffer/ethanol.

**FIGURE 6:**
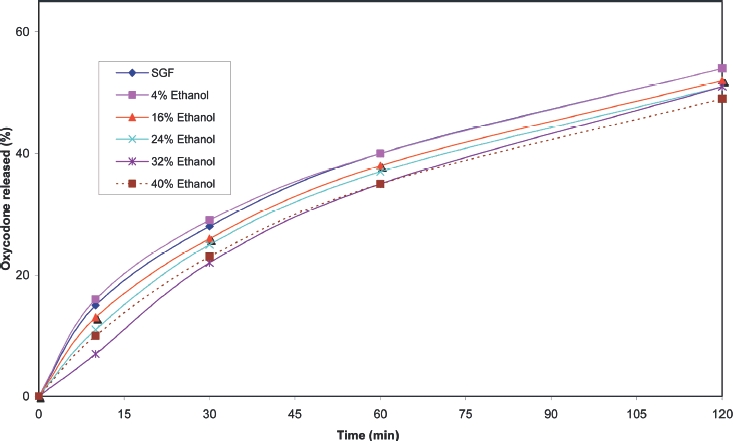
Cumulative release of oxycodone from 80 mg *OxyContin* tablets in simulated gastric fluid/ethanol.

**FIGURE 7:**
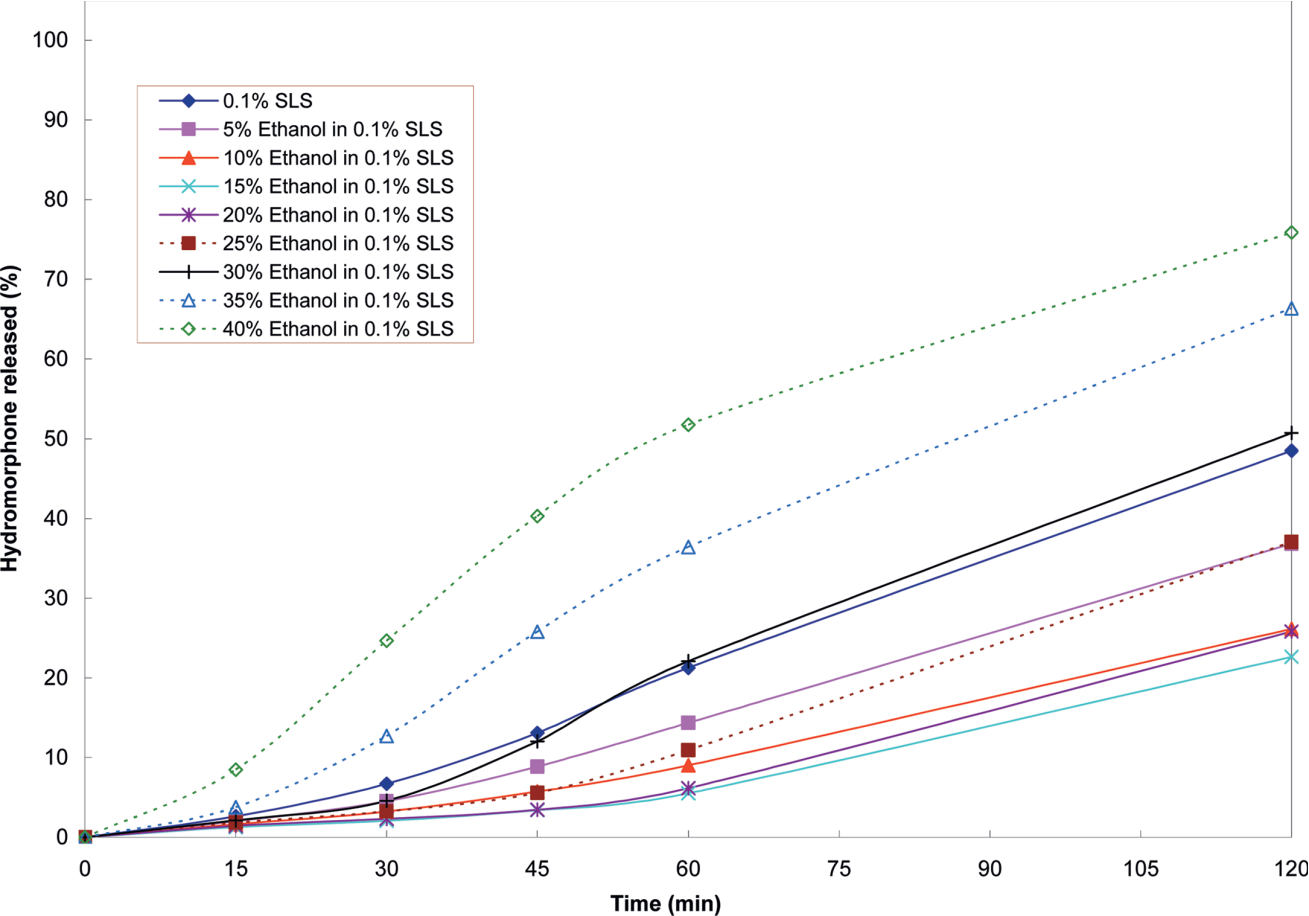
Cumulative release of hydromorphone from *Palladone* SR capsules in 0.1% w/v sodium lauryl sulphate/ethanol.

**FIGURE 8:**
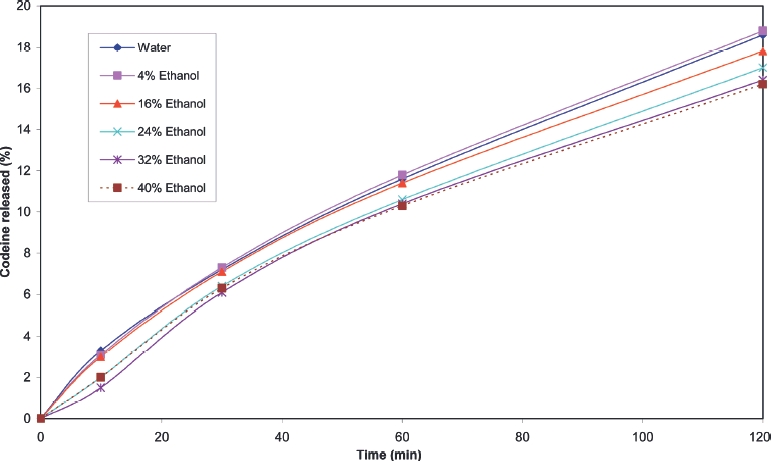
Cumulative release of codeine from Codeine *Contin* 100 mg tablets in water/ethanol.

**FIGURE 9:**
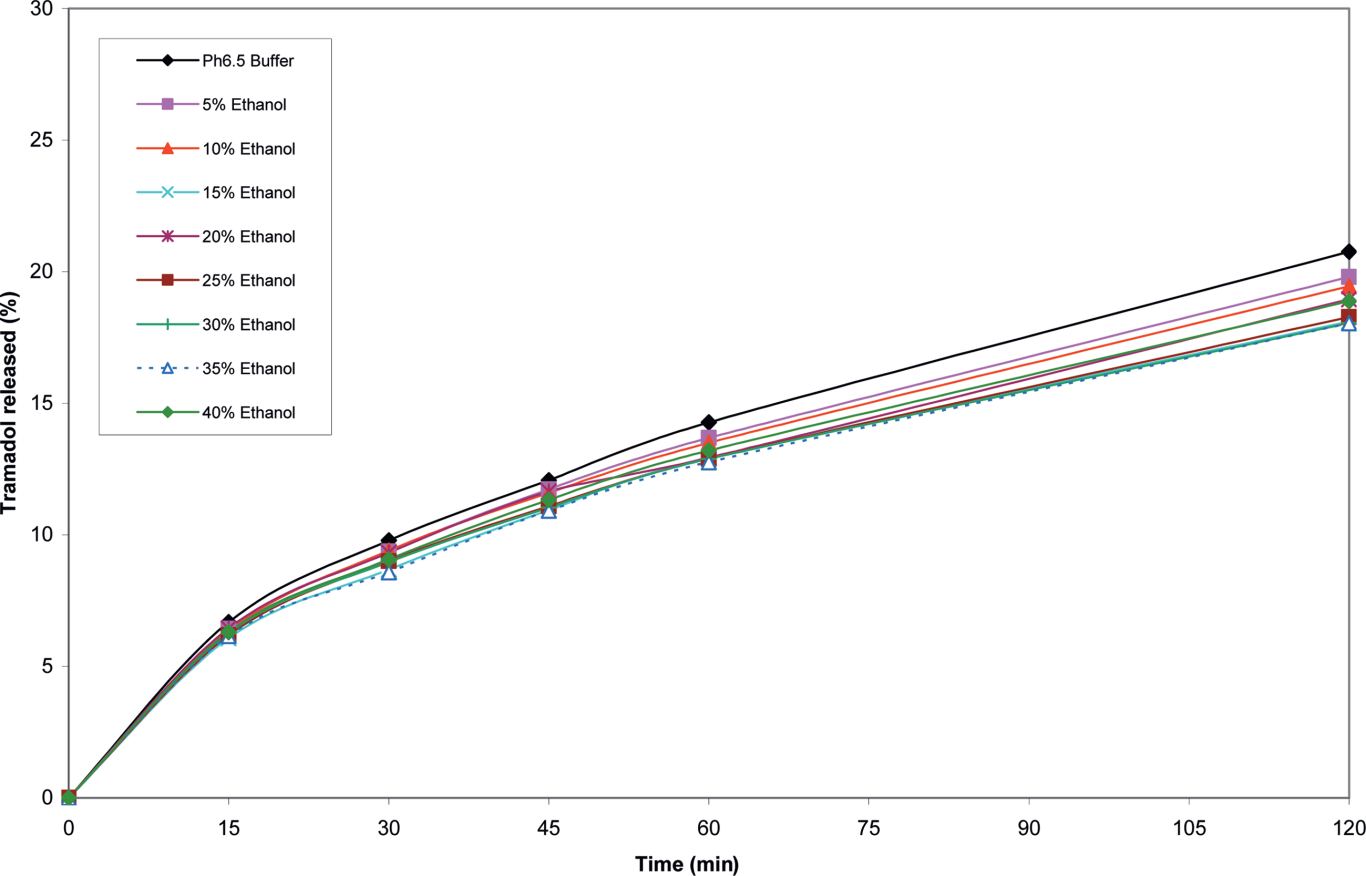
Cumulative release of tramadol from tramadol 200 mg b.d. tablets in pH 6.5 buffer/ethanol.

**FIGURE 10:**
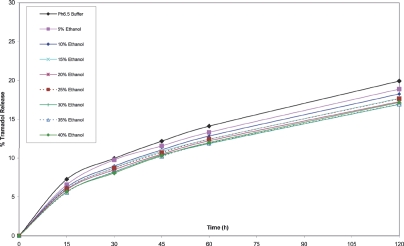
Cumulative release of tramadol from tramadol 400 mg o.d. tablets in pH 6.5 buffer/ethanol.

Cumulative release profiles for each of the products tested are shown in Figures 2 to 10. The results for most of the products indicated a negligible effect of ethanol on the release of the active ingredient. *Palladone* SR capsules, which utilise a coated bead technology, demonstrate a distinct biphasic effect as a function of ethanol concentration–the initial drug release rate decreased as the ethanol concentration was increased from 5 to 15% v/v then increased to control values at 30% v/v with a further increase compared to control values at 35–40% v/v. To provide confidence in this observation, the experiment was repeated using a different batch of Palladone SR capsules and taking the mean result for 6 separate capsules at alcohol concentrations of 10, 30, and 40% v/v. The effect of ethanol on the pattern of drug release was confirmed, and the maximum coefficient of variation of drug release within unit doses across all sampling points was 3%.

## DISCUSSION

Experience with the once-daily hydromorphone preparation has highlighted the potential for ethanol to alter the retardation properties of prolonged-release formulations. The exact effect will depend upon gastric emptying time, the absorption and metabolism of ethanol in the stomach, fluid secretion into the stomach and the nature of the formulation.

Normally, the gastric emptying of liquids is rapid with a half time of 12 min, such that 95% is emptied in 1 h (Petring & Blake).

Levitt et al. (1997) found that, in fasted individuals, 90% of a 0.15 g/kg dose of ethanol had emptied from the stomach by 20–30 min, with the remainder being absorbed from the stomach in this time. In the fed state a half-time of 50 min for liquid emptying was reported, with about 70% of the ethanol passing into the duodenum after 2h and 30% absorbed from the stomach.

Franke et al. (2004) reported that pure ethanol inhibited gastric emptying. Mean half-emptying times of 22.6, 22.7 and 27.8 min, with ethanol solutions of 4%, 10% and 40% respectively were significantly longer than those after ingestion of water (14.6 min). Moreover, the inhibitory effects of beer (half emptying time of 39.3 min) and red wine (half emptying time of 72.6 min), but not of whisky (half emptying time of 26.4 min), were greater than those of their comparable ethanol concentrations. This suggested that the caloric content and non-ethanolic ingredients in ethanolic beverages produced by fermentation, but not those produced by distillation, were also partly responsible for this effect.

Oneta et al. (1998) indicate that a delay in gastric emptying increases exposure of ethanol to gastric ethanol dehydrogenase, thereby increasing its metabolism.

Basal gastric secretion rates of up to 3 L/day (125 mL/h) have been reported Freitas (1999). Thus, there will be a significant dilution of any ethanol in the stomach with time. It should also be noted that ethanol undergoes rapid absorption once it is emptied from the stomach and this will curtail any continuing effect on drug release from a prolonged-release formulation (Roine et al., 1991).

Recognizing the above variables, it was considered that in vitro dissolution studies over 2 h with ethanol concentrations of up to 40%v/v should afford confidence with regard to the likelihood of altered release rate or dose-dumping consistent with the most extreme conditions likely to be encountered in vivo.

Of the products tested in this study, only one (*Palladone* SR capsules) indicated a clear increase in dissolution rate in the first hour in the presence of high concentrations of ethanol. Thereafter, hydromorphone was released at a rate consistent with that of the control, as demonstrated by the parallel slopes of the release curves.

Simulation of the in vivo impact of this on the in vivo kinetics of hydromorphone (unpublished) indicated that any increase in peak plasma hydromorphone concentration arising from a 2 h exposure to 40% ethanol would be expected to be of the order of 15–30%. This change is similar in magnitude to that commonly observed when some prolonged-release formulations are compared under fed and fasting conditions. Therefore, such an increase would not be expected to be of clinical significance.

Figure 1 shows clearly that with the hydromorphone once a day formulation that was withdrawn in the USA, ethanol in concentrations up to 40%v/v had a major effect in increasing the rate of drug release, producing a ‘dose dumping’ consistent with the subsequent in vivo findings. This particular formulation contains significant quantities of release controlling excipients that are soluble in 40% v/v ethanol but not in water, although the effect of ethanol may depend on other formulation factors as well as discussed below.

The dissolution conditions used routinely for quality control of the release properties of the products that we have investigated differ in terms of pH and dissolution media (Table 2). However, in order to provide a standardised testing approach, regulatory guidance on the appropriate conditions and duration of testing is encouraged.

The products investigated cover a variety of prolonged-release formulation technologies and utilise a broad spectrum of release-controlling excipients. The results have shown that the prolonged-release mechanisms of the products remain intact under the testing conditions. When extrapolated to the in vivo situation, the data indicate that there is no risk of a potentially dangerous dose-dumping. It is believed that the experimental conditions selected, which are expected to be representative of the most extreme conditions likely to be encountered in vivo, were justified by our understanding of gastric conditions and the fate of ethanol in the stomach.

Analysis of these data and unpublished confidential material indicates that there is no clear correlation between the ethanol solubility of the ingredients and the ethanol susceptibility of formulations, rather there may be complex interactions between active and inactive ingredients and dosage form design. Therefore, it is proposed that all prolonged-release medicinal products should be subjected to in vitro testing during formulation development to ensure their robustness to the effects on drug release when consumed with alcoholic beverages. This is of particular concern with preparations containing drugs with a narrow therapeutic index or drugs such as strong opioids where careful titration of dosage is necessary. A standardized approach to testing needs to be agreed and adopted internationally by the pharmaceutical industry and regulatory authorities.
